# Temperature and current flow effects of different electrode placement in shoulder capacitive-resistive electric transfer applications: a cadaveric study

**DOI:** 10.1186/s12891-020-03918-7

**Published:** 2021-02-04

**Authors:** Jacobo Rodríguez-Sanz, Carlos López-de-Celis, César Hidalgo-García, Max Canet-Vintró, Pablo Fanlo-Mazas, Albert Pérez-Bellmunt

**Affiliations:** 1 Universitat Internacional de Catalunya. Actium functional anatomy group. Faculty of Medicine and Health Sciences, Barcelona, Spain; 2 Fundació Institut Universitari per a la recerca a l’Atenció Primària de Salut Jordi Gol i Gurina, Barcelona, Spain; 3 Facultad de Ciencias de la Salud de la Universidad de Zaragoza, Unidad de Investigación en Fisioterapia, c/ Domingo Miral s/n, 50009 Zaragoza, Spain

**Keywords:** Supraspinatus tendon, Cadaver, CRet, Shoulder, Glenohumeral capsule, Physical therapy

## Abstract

**Background:**

Impingement syndrome is currently estimated to represent 60% of all shoulder pain disorders. Capacitive-Resistive electric transfer therapy is aimed to provoke temperature and current flow changes in superficial and deep tissues. This in vitro study has evaluated the variation of temperature and current flow in the shoulder tissues during two different areas of application of the movable capacitive-resistive electric transfer electrode.

**Methods:**

A cross-sectional study designed, five fresh cryopreserved cadavers (10 shoulders) were included in this study. Four interventions (capacitive and resistive modes; low- and high-power) were performed for 5 min each by a diathermy “T-Plus” device in two shoulder regions: postero-superior and antero-lateral. Supraspinatus tendon, glenohumeral capsule and superficial temperatures were recorded at 1-min intervals and 5 min after treatment.

**Results:**

A statistically significant difference was found only for the superficial area and time interaction, with high power-resistive application at the postero-superior shoulder area (*P*< 0.035). All the applications showed a 5 min after treatment temperature increase compared with the basal data, in all the application points. Superficial temperature in the high power-resistive application showed the greatest percent increase (42.93% ± 22.58), followed by the temperature in the tendon area with the same high power-resistive application (22.97% ± 14.70). The high power-resistive application showed the greatest percent of temperature increase in the applications, reaching 65.9% ± 22.96 at 5-min at the superficial level, and 32% ± 24.25 at 4-min at the level of the supraspinatus tendon. At the capsule level, high power-resistive was also the application that showed the greatest percent of increase, with 21.52% ± 16.16. The application with the lowest percent of temperature increase was the low power-capacitive, with a mean value of 4.86% at supraspinatus tendon level and 7.47% at capsular level.

**Conclusion:**

The shoulder postero-superior or antero-lateral areas of application of capacitive-resistive electric transfer did not cause statistically significant differences in the temperature changes in either supraspinatus tendon or glenohumeral capsule tissues in cadaveric samples. The high power-resistive application in the postero-superior area significantly increased superficial temperature compared with the same application in the antero-lateral position area.

## Background

Impingement syndrome is currently estimated to represent 44–65% of all shoulder pain disorders [1]. The most normal causes of symptoms are extrinsic causes such as work and sport overload [2]. These factors can cause the appearance of various types of rotator cuff pathologies, especially in athletes and manual labourers [1]. Usually affecting the supraspinatus tendon, these pathological conditions have been called “impingement” and have produced strong interest in treatment methods [2]. Another important disorder that affects this population is capsulitis, especially postoperative capsulitis from interventions related to rotator cuff repair and shoulder arthroplasty [3]. Lengthy immobilisation or surgical entry sites can cause excess fibrous tissue, limiting movement and producing symptoms in tendinous areas [4].

The increase in the concentration of type I collagen that is seen in capsular disorders, such as adhesive capsulitis, leads to decreased range of movement [3]. Increasing the temperature by 1 °C can have several effects in the human body, such as changes in nerve conduction speed, enzyme activity and oxyhaemoglobin release [5–8]. Tissue hypoxia produces tissue fibrosis and the generation and release of algesic substances, which cause pain, muscle spasms and joint rigidity [9]. An increase in temperature can improve oxygenated haemoglobin saturation [9].

Likewise, it is known that vascular supply affects tendon tissue repair [10]. Animal studies have demonstrated that when the blood supply in the tendon is interrupted, there are changes such as separation of the tendon fascicles, loss of the normal properties of the tenocytes in the interfascicular spaces (which shorten or degenerate) and collagen fibre fragmentation. It has also been shown that the changes observed in chronic degenerative tendon disorders are the same as those produced when blood supply to the rabbit tendon is altered [11]. Consequently, vascular supply is one of the key factors in the approach to muscle and tendon tissues.

Capacitive-resistive electric transfer (CRet) is usually used to treat muscle, joint and tendon injuries in the areas of traumatology and sports [12]. CRet is a non-invasive electrothermal therapy classified as deep thermotherapy; it is based on applying electric flow in the radiofrequency range of 300 kHz-1.2 MHz [7]. In contrast to superficial thermotherapy, which has a very limited capacity to reach muscle tissue [13], CRet can generate heat in deep muscle tissue, improving haemoglobin saturation [9]. The physiological effects of this type of therapy stem from applying an electromagnetic field of approximately 0.5 MHz to the human body. The effects attributed to this technique include increasing blood circulation and deep and superficial temperature, vasodilation, lymphatic effects and raising cell proliferation [14]. It has been observed that the increased blood perfusion is linked to the temperature increase, but other effects –such as the cell proliferation– seem to be mainly related to current flow [14, 15]. Cell proliferation has been shown to begin from a current flow of 0.00005 A per square millimetre [15].

The temperature increase in the tissues that the CRet device generates is a physical reaction to the current flow (Joule effect) [7, 16]. Although there are clinical studies that support this mechanism, the amounts of energy and current that should be transferred to obtain the desired temperature increase in structures such as the supraspinatus tendon and the joint capsule are still unknown. In addition, controlling these reactions is still based, to a great extent, on empirical experience from therapists and commercial brand protocols [9, 12, 17].

A single study analysing temperature and current flow changes in the Achilles tendon and the myotendinous junction of the gastrocnemius muscles in cadavers has been found [7]. However, we have not found any studies on these measurements in shoulder structures. Furthermore, no study has been found that evaluates whether varying the placement of the application electrode produces relevant differences in the target tissue during shoulder treatment.

The hypothesis was that alternating electrode position would generate the same current flow and differences in temperature changes in both superficial and deep tissues of the shoulder during the application of capacitive and resistive CRet protocols in cadaveric specimens.

## Methods

### Aim

The main objective of this study was to verify whether changing the position of the electrodes modified current flow and temperature in superficial and deep shoulder tissues by measuring these factors invasively during the application of capacitive and resistive CRet protocols in cadaveric specimens.

### Study design

A cross-sectional study was designed to establish the effect of transferring electrical capacitive/resistive energy from the Wintecare T-Plus device on the temperature and current flow in the shoulder area (superficial, supraspinatus tendon and glenohumeral capsule) in cadaveric specimens. The body donation programme at the Faculty of Medicine and Health Sciences of the Universitat Internacional de Catalunya (Spain) provided all the samples. The study was approved by the “Comitè d’Ètica de Recerca” (CER) research ethics committee at the Universitat Internacional de Catalunya (Reference number CB12020).

### Cadaveric specimens

The sample was composed of 5 complete, fresh, cryopreserved cadavers (3 men and 2 women; 10 limbs). The cadavers were stored 3 weeks at − 14 °C, then they were kept at 3 °C 2 days before the testing and brought to room temperature a day before the study. The study was done progressively as the body donors arrived. Mean cadaver age was 80.6 ± 14.6 years. None of the cadaveric samples used in this study had evidence of trauma or surgical scars on the limbs.

### Intervention

For better understanding of the temperature changes and electric current flow in conditions similar to rehabilitation treatments, we adapted a power threshold similar to that normally used during treatments on real patients with the T-Plus model [7, 18] but without the possibility of receiving patient’s feedback. This is based on the power level that the therapist can easily identify and control during the therapy, and the watts (absorbed power) that the device samples during application. The power range of a T-Plus device varies from 1 to 300 watts in resistive mode and from 1 to 450 V-ampere (VA) in capacitive mode [7].

Two thresholds for high and low power were identified, based on the empirical evidence that the therapist applies clinically when inducing a thermal or non-thermal reaction, respectively, is desired. The power depends on the protocol used, which is a function of the area to be treated. In the shoulder area, high power-capacitive (HPC) application is defined as applying 130 VA in capacitive mode, and high power-resistive (HPR), as applying 100 watts in resistive mode; for the low power applications, low power-capacitive (LPC) is defined as applying 40 VA and low power-resistive (LPR), 20 watts. In comparison with treatments typically used in real patients, these low power thresholds respect the limit of 0.3 A, while the high power thresholds are above 0.3 A, so thermal effects are expected [7].

**Fig. 1:**
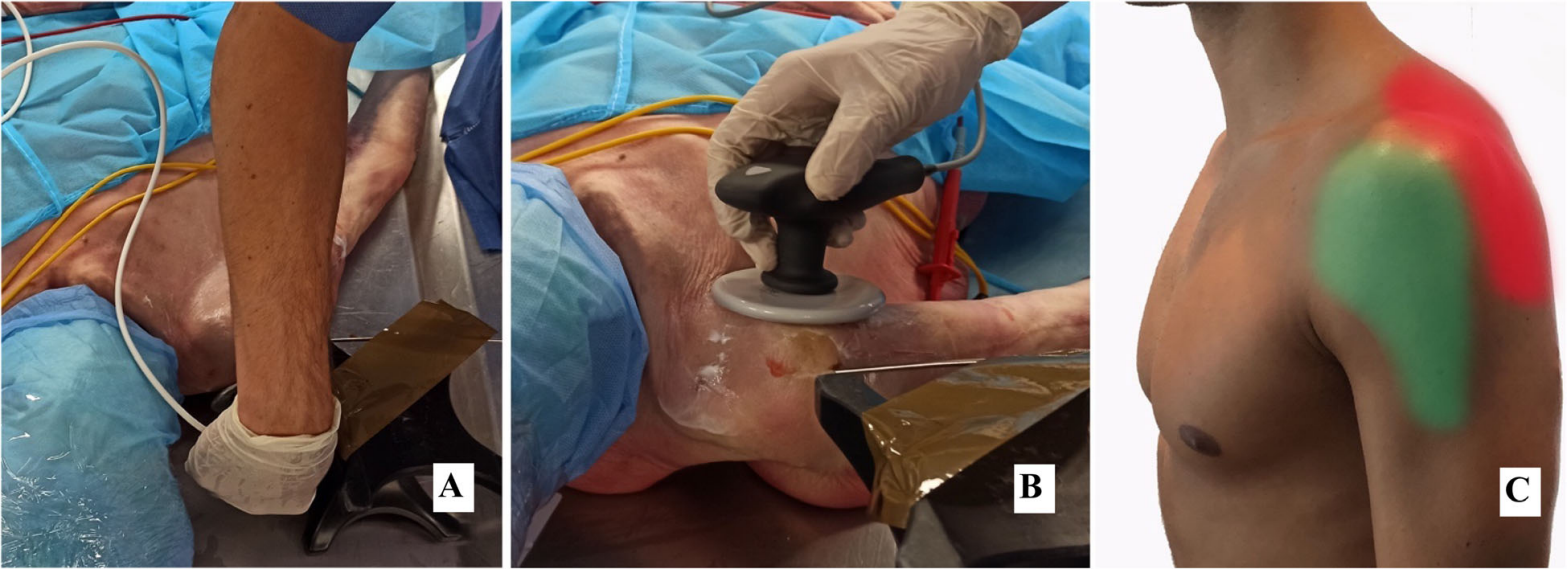
Application of the interventions in the different electrode localization in the shoulder region. **a** Postero-superior application. **b** Antero-lateral application. **c** Application areas (Green = Antero-lateral; Red = Postero-superior). Experimental Procedure

Four interventions (HPC, HPR, LPC and LPR) of 5 min each were performed, with the return electrode on the lower back of the cadaveric specimen and the movable electrode on the antero-lateral shoulder area. The same 4 interventions were applied changing the movable electrode position to the postero-superior part of the shoulder area, near the acromioclavicular joint. Dynamic movements similar to those used with real patients were made using constant pressure. The treatments were given by a Tecar-certified physiotherapist with 5 years of experience in the use of the T-Plus (Fig. 1).

### Experimental procedures

Each cadaver was placed in a supine position with the forearm in a neutral pronation-supination position, the elbow extended and the shoulder in neutral flexion-extension.

The order of the 8 treatment protocols and the arm of each cadaver were randomly assigned before the study. An external researcher randomised these 2 factors using the computer programme “random.org”. Before applying each treatment, it was ensured that the basal temperature of each cadaver had returned to the initial values.

Before beginning the CRet application, all instruments used were verified to have a calibration certificate. Hart Scientific PT25 5628–15 temperature devices were used to measure the tendinous and capsular temperature (°C) of the shoulder. A “Thermocomed” digital thermometer was used to measure the superficial skin temperature in the shoulder area. Thermocouples were placed under ultrasound guidance (US Aloka ProSound C3 15.4″) with a high-frequency linear transductor (USTTL01, 12 L5) in the middle of the supraspinatus tendon and in the glenohumeral joint capsule by a researcher expert in the use of the instrument. The return electrode of the T-Plus was placed on the lower back of the cadavers. Each treatment was performed with the T-Plus movable electrode on the previously-explained treatment areas for 5 min. The initial superficial temperatures and those registered by the invasive temperature monitors were measured. These measurements were registered at intervals of 1 min during the 5-min treatment period and then at 5 min after the end of each treatment. Prior to treatment, the impedance was always recorded (Fluke 8846A Digital Multimeter) to guarantee that the values marked by the Wintecare T-Plus device were correct. The current flow existing at that time was also calculated for each application, using the mean voltage divided by the initial impedance.

### Statistical analysis

Statistical analysis was performed with the SPSS Statistics version 22.0 programme. Normal distribution was calculated using the Shapiro-Wilk test (*P*> 0.05). The mean, standard deviation and difference between applications were calculated, as well as the percent of temperature increase.

Two-way repeated measures analysis of variance (ANOVA) was used for the comparative analysis. Statistical significance was set to *p*< 0.05.

## Results

The descriptive values of the temperature recorded during the various applications, in the different superficial measurement intervals (Table 1), in the supraspinatus tendon (Table 2) and in the joint capsule were calculated (Table 3). A statistically-significant difference was found only for the area and time interaction, with HPR application at the postero-superior level (*P*< 0.035).

**Table 1:** Descriptive values of the temperature recorded during the various applications in the different superficial measurement intervals

	Superficial	Baseline	1 min	2 min	3 min	4 min	5 min	5 min after treatment	ANOVA F; *p* Value
HPC	Antero-Lateral	21.09 ± 2.10	25.35 ± 2.31	27.19 ± 2.74	28.09 ± 3.08	29.77 ± 3.15	31.50 ± 4.11	22.97 ± 2.07	*F=*0.871 *p*< 0.522
	Postero-Superior	22.32 ± 2.33	27.44 ± 3.81	29.84 ±5.18	31.53 ± 6.20	34.06 ± 6.58	34.14 ± 6.41	25.51 ± 3.37	
	Difference	1.23 ± 2.62	2.09 ± 3.75	2.65 ± 5.10	3.44 ± 6.37	4.29 ± 6.64	2.64 ± 6.94	2.54 ± 3.58	
LPC	Antero-Lateral	20.63 ± 3.09	21.88 ± 1.55	22.07 ± 1.81	22.74 ± 2.05	23.24 ± 1.66	23.93 ± 2.34	20.94 ± 2.37	*F=*1.073 *p*< 0.390
	Postero-Superior	22.48 ± 2.52	25.21 ± 3.62	25.87 ± 4.24	25.65 ± 4.44	25.69 ± 4.70	22.83 ± 14.56	22.67 ± 2.09	
	Difference	1.85 ± 1.60	3.33 ± 3.17	3.80 ± 3.48	3.91 ± 3.45	3.45 ± 3.69	1.10 ± 14.17	1.73 ± 1.63	
HPR	Antero-Lateral	19.69 ± 2.48	24.81 ± 5.60	25.86 ± 5.96	27.07 ± 5.71	27.91 ± 5.89	29.09 ± 6.50	25.41 ± 3.86	*F=*2.469 ***p<*** **0.035**
	Postero-Superior	21.46 ± 2.77	28.76 ± 6.47	31.10 ± 7.23	33.22 ± 8.16	35.44 ± 9.23	35.65 ± 8.02	30.75 ± 6.82	
	Difference	1.77 ± 2.51	3.95 ± 5.46	5.24 ± 7.50	6.15 ± 6.66	7.53 ± 6.61	6.56 ± 6.72	5.34 ± 3.80	
LPR	Antero-Lateral	20.67 ± 2.25	21.78 ± 2.11	22.91 ± 3.05	23.46 ± 3.54	23.42 ± 2.51	23.96 ± 3.51	21.62 ± 3.39	*F=*1.093 *p*< 0.378
	Postero-Superior	21.87 ± 2.37	24.22 ± 3.27	25.21 ± 3.82	25.72 ± 4.79	26.10 ± 4.22	26.91 ± 5.38	23.84 ± 3.67	
	Difference	1.20 ± 2.06	2.44 ± 2.36	2.30 ± 1.77	2.26 ± 3.12	2.68 ± 2.60	2.95 ± 3.78	2.22 ± 2.35	

Abbreviations: *HPC* High Power Capacitive, *LPC* Low Power Capacitive, *HPR* High Power Resistive, *LPR* Low Power Resistive

**Table 2:** Descriptive values of the temperature recorded during the various applications in the supraspinatus tendon

	Supraspinatus tendon	Baseline	1 min	2 min	3 min	4 min	5 min	5 min after treatment	ANOVA F; *p* Value
HPC	Antero-Lateral	21.44 ± 2.82	22.72 ± 2.46	23.39 ± 2.34	23.99 ± 2.58	24.42 ± 2.49	24.99 ± 2.96	24.71 ± 2.07	*F=*0.180 *p*< 0.981
	Postero-Superior	23.52 ± 1.32	24.78 ± 1.74	25.52 ± 2.06	26.28 ± 2.44	26.91 ± 2.55	27.40 ± 2.79	27.29 ± 2.31	
	Difference	2.08 ± 2.85	2.06 ± 3.05	2.13 ± 3.46	2.29 ± 4.33	2.49 ± 4.42	2.41 ± 5.05	2.58 ± 3.49	
LPC	Antero-Lateral	22.24 ± 2.73	22.93 ± 2.55	23.07 ± 2.72	23.30 ± 2.33	23.57 ± 2.08	23.68 ± 1.97	23.86 ± 2.36	*F=*0.769 *p*< 0.597
	Postero-Superior	24.05 ± 2.03	25.11 ± 2.05	25.54 ± 2.16	25.81 ± 2.39	25.99 ± 2.49	26.24 ± 2.54	25.99 ± 1.55	
	Difference	1.81 ± 1.92	2.18 ± 2.01	2.47 ± 2.48	2.51 ± 2.41	2.42 ± 2.47	2.56 ± 2.66	2.13 ± 1.97	
HPR	Antero-Lateral	21.14 ± 2.75	26.30 ± 1.93	27.02 ± 1.75	28.96 ± 2.15	30.12 ± 2.03	31.63 ± 3.10	28.01 ± 1.87	*F=*1.075 *p*< 0.389
	Postero-Superior	23.13 ± 2.55	28.27 ± 5.32	28.13 ± 4.50	28.87 ± 5.06	30.48 ± 6.37	30.64 ± 5.94	28.35 ± 4.04	
	Difference	1.99 ± 1.88	1.97 ± 4.19	1.11 ± 4.54	−0.09 ± 4.78	0.36 ± 6.69	−0.99 ± 7.33	0.34 ± 3.80	
LPR	Antero-Lateral	21.34 ± 3.66	22.69 ± 3.14	23.15 ± 3.07	23.68 ± 2.90	24.05 ± 3.03	24.41 ± 3.09	24.23 ± 2.99	*F=*1.636 *p*< 0.155
	Postero-Superior	24.14 ± 1.94	25.65 ± 1.85	25.77 ± 1.84	26.04 ± 1.60	26.50 ± 2.19	26.60 ± 2.04	25.82 ± 1.69	
	Difference	2.80 ± 2.75	2.96 ± 2.97	2.62 ± 3.23	2.36 ± 3.41	2.45 ± 3.18	2.19 ± 3.53	1.59 ± 2.48	

Abbreviations: *HPC* High Power Capacitive, *LPC* Low Power Capacitive, *HPR* High Power Resistive, *LPR* Low Power Resistive

**Table 3:** Descriptive values of the temperature recorded during the various applications in the glenohumeral capsule

	Joint Capsule	Baseline	1 min	2 min	3 min	4 min	5 min	5 min after treatment	ANOVA F; *p* Value
HPC	Antero-Lateral	19.02 ± 2.21	20.67 ± 3.62	20.78 ± 3.25	20.88 ± 3.27	21.13 ± 3.42	21.82 ± 4.27	20.01 ± 2.43	*F=*0.567 *p*< 0.755
	Postero-Superior	19.94 ± 1.68	21.64 ± 3.15	21.93 ± 3.27	22.22 ± 3.52	22.32 ± 3.52	22.45 ± 3.55	21.23 ± 1.28	
	Difference	0.92 ± 1.53	0.97 ± 1.82	1.15 ± 1.84	1.34 ± 1.96	1.19 ± 2.05	0.63 ± 2.86	1.22 ± 1.72	
LPC	Antero-Lateral	19.14 ± 2.97	21.24 ± 5.30	20.62 ± 3.46	20.84 ± 3.53	21.04 ± 3.60	21.13 ± 3.71	20.30 ± 2.69	*F=*0.859 *p*< 0.531
	Postero-Superior	20.33 ± 1.74	21.12 ± 1.91	21.27 ± 1.89	21.44 ± 1.98	21.40 ± 1.92	21.41 ± 1.94	21.24 ± 1.63	
	Difference	1.19 ± 1.75	−0.12 ± 3.96	0.65 ± 2.52	0.60 ± 2.78	0.36 ± 2.67	0.28 ± 2.77	0.94 ± 2.10	
HPR	Antero-Lateral	19.04 ± 4.23	20.72 ± 3.73	21.30 ± 3.21	21.56 ± 3.27	22.10 ± 3.35	22.08 ± 3.55	20.69 ± 2.67	*F=*0.550 *p*< 0.768
	Postero-Superior	19.04 ± 2.34	21.42 ± 3.97	21.43 ± 2.82	21.42 ± 2.38	22.33 ± 3.71	23.22 ± 4.95	21.68 ±3.14	
	Difference	0.00 ± 2.97	0.70 ± 3.53	0.13 ± 2.18	−0.14 ± 2.15	0.23 ± 3.35	1.14 ± 4.64	0.99 ± 1.68	
LPR	Antero-Lateral	18.48 ± 2.77	19.60 ± 2.27	19.71 ± 2.26	19.94 ± 2.13	20.17 ± 2.05	20.36 ± 2.04	20.37 ± 2.15	*F=*0.659 *p*< 0.683
	Postero-Superior	19.70 ± 2.96	20.50 ± 2.39	20.74 ± 2.19	20.92 ± 2.08	21.05 ± 1.98	21.16 ± 1.89	21.26 ± 1.94	
	Difference	1.22 ± 2.11	0.90 ± 1.99	1.03 ± 1.91	0.98 ± 1.72	0.88 ± 1.67	0.80 ± 1.58	0.89 ± 1.60	

Abbreviations: *HPC* High Power Capacitive, *LPC* Low Power Capacitive, *HPR* High Power Resistive, *LPR* Low Power Resistive

Current flow during antero-lateral application was 0.27±0.01 A (HPC 130 VA); 0.15±0.01 A (LPC 40 VA); 0.40±0.01 A (HPR 130 W); and 0.22±0.01 (LPR 30 W). In the postero-superior application, it was 0.21±0.01 A (HPC 130 VA); 0.14±0.02 A (LPC 40 VA); 0.45±0.01 A (HPR 130 W); and 0.31±0.02 (LPR 30 W).

All the applications showed a 5 min after treatment temperature increase compared with the basal data, in all the application points. Superficial temperature in the HPR application showed the greatest percent increase (42.93% ± 22.58), followed by the temperature in the tendon area with the same HPR application (22.97% ± 14.70).

The HPR application showed the greatest percent of temperature increase in the applications, reaching 65.9% ± 22.96 at Minute 5 at the superficial level, and 32% ± 24.25 at Minute 4 at the level of the supraspinatus tendon. At the capsule level, HPR was also the application that showed the greatest percent of increase, with 21.52% ± 16.16.

The application with the lowest percent of temperature increase was the LPC, with a mean value of 4.86% at supraspinatus tendon level and 7.47% at capsular level.

## Discussion

CRet therapy is a technique whose use is growing steadily in clinical treatments. However, a significant lack of studies using this tool currently keeps us from being able to evaluate its effectiveness or better know its capacities and limitations. One of the main questions posed using this type of therapy is whether we get the same results applying the same dose in different zones in the same area. To date, this is the first study to compare the thermal and current flow effects from CRet therapy in the shoulder area, comparing applications (HPR, LPR, HPC and LPC) in different zones (antero-lateral and postero-superior) of the same area.

Our study results suggest that there are no significant differences in temperature of the glenohumeral joint capsule and the supraspinatus tendon between the different zones (postero-superior and antero-lateral) among any of the applications performed (HPR, LPR, HPC and LPC) in cadavers. The only significant difference found was the superficial temperature during HPR application, with a greater temperature increase being produced with postero-superior application.

All the applications, whether antero-lateral or postero-superior, generate a current flow above 0.03 A. These findings indicate that all the applications would be capable of causing cell proliferation in live subjects in the structures in which it is being measured (supraspinatus tendon and joint capsule) [7, 14, 15]. Clinically, this cell proliferation has been linked to increased blood supply and to tendinous tissue repair [10].

There is a single study that has analysed changes in temperature and current flow in cadavers in the Achilles tendon and the myotendinous junction of the gastrocnemius muscles [7]. This study also observed current flows higher than the minimum to cause cell proliferation and found a thermal increase at the level of the monitored and deep structures similar to those found in our study. However, neither in that study or any other are there comparisons about the location of the applications by zones. Considering the extrapolation of this in vitro thermal increase into an in vivo clinical situation, the absolute temperature values throughout our in vitro study will not probably be present [19] in a living body as vasodilation and increased blood flow will appear to keep homeostasis preventing excessive tissue warming and damage. This vasodilation and the increased blood flow is a functional body response that will ease the cooling of the tissues by convection. Presumably, on one hand, the increased temperature will increase the cellular and metabolic activity, the extensibility of the collagen fibers and the nerve conduction velocity, alter the vascular and synovial viscosity and will reduce the muscle tone in the treated tissues [13]. On the other hand, the increase of blood flow will ease the drainage and elimination of waste products in the tissues with oedema. However, all this potentially beneficial events in the living body need to be validated in shoulder pathologies like rotator cuff or capsular shoulder pathology.

Capacitive applications are concentrated in the tissues containing more electrolytes (muscles and soft tissues); conversely, resistive applications are concentrated in the structures with the greatest resistances (bones, tendons and joints) [20, 21]. Capacitive applications typically penetrate more deeply in the skin perpendicularly to the deep structures, while in resistive applications, in contrast, the current conversely searches for “the shortest path” to the return electrode through the resistance of the tissues and structures [20, 21].

In this study, no differences between the antero-lateral and postero-superior applications have been found. These results might be due to the fact that the two zones are very close to each other and the mechanism of action of the capacitive and resistive applications was not altered. However, the lack of evidence prevents us from stating this conclusively. On the other hand, these results might indicate that applying different CRet dosages at the shoulder level could produce similar changes in adjacent zones. This would be useful in clinical practice to treat patients with symptoms by using adjacent areas that are less symptomatic and have an effect on the affected tissue zone.

The lack of evidence on CRet therapy in both the shoulder area and in applying the therapy in different zones in the same area makes new studies necessary. These studies should compare applications in areas further apart to ascertain whether the effect found in this study is similar or, on the contrary, different.

## Conclusion

Varying the electrode position does not cause statistically-significant differences in the temperature changes in either superficial or deep shoulder tissues in cadaveric samples. The application of postero-superior HPR was the only one that significantly increased superficial temperature compared with the same application in antero-lateral position.

### Study limitations

The limitations of this study are discussed in this section. Our study uses cadaveric specimens in which there is no thermoregulation or active blood circulation. This factor has probably impacted the temperature changes. In live subjects, the thermoregulation effect exists in the body; it controls heat dissipation, so the temperature increases in such subjects would predictably have been lower. This effect helps to avoid undesired hyperthermia during treatment in real patients [9]. Another limitation is that, although the cadavers were cryopreserved, the muscle and tendinous tissue properties might vary from those of live subjects. In addition, the average age of the corpses used was relatively high. Despite these limitations, the authors consider that the use of donated bodies has made it possible to ascertain how the various CRet applications impact temperature and current flow values in structures typically affected, which is ethically inviable in live subjects. As discussed previously, the lack of evidence on these procedures in cadavers makes extrapolating the results to clinical practice complicated.

## Data Availability

The datasets used and/or analysed during the current study are available from the corresponding author on reasonable request.

## Abbreviations

CRet: Capacitive-resistive electric transfer
HPC: High-power capacitive
LPC: Low-power capacitive
HPR: High-power resistive
LPR: Low-power resistive
